# Sodium Content and Labelling Completeness of Packaged Foods and Beverages in Kenya

**DOI:** 10.3390/nu13041385

**Published:** 2021-04-20

**Authors:** Rhoda Ndanuko, Damian Maganja, Alex Kibet, Daisy H. Coyle, Judith Kimiywe, David Raubenheimer, Matti Marklund, Jason H. Y. Wu

**Affiliations:** 1 The George Institute for Global Health, University of New South Wales, Sydney, NSW 2042, Australia; dcoyle@georgeinstitute.org.au (D.H.C.); mmarklund@georgeinstitute.org.au (M.M.); jwu1@georgeinstitute.org.au (J.H.Y.W.); 2 Department of Food, Nutrition and Dietetics, Kenyatta University, Nairobi 00100, Kenya; alexkibet.ak5@gmail.com (A.K.); kimiywe.judith@ku.ac.ke (J.K.); 3 Charles Perkins Centre, The University of Sydney, Sydney, NSW 2006, Australia; david.raubenheimer@sydney.edu.au; 4 Department of Epidemiology, Johns Hopkins Bloomberg School of Public Health, Baltimore, MD 21205, USA; 5 Department of Public Health and Caring Sciences, Uppsala University, 751 22 Uppsala, Sweden

**Keywords:** Kenya, nutrition labelling, salt, sodium, packaged foods, processed foods

## Abstract

Increased consumption of unhealthy processed foods, particularly those high in sodium, is a major risk factor for cardiovascular diseases. Nutrition information on packaged foods can help guide consumers toward products with less sodium, however the availability of nutrition information on foods sold in Kenya is currently unknown. The aims of this study were to estimate the proportion of packaged foods and beverages displaying nutrition information for sodium and determine the amount of sodium in packaged foods available for sale in Kenya. Data was collected in 2019 from five retail supermarkets in Nairobi. The availability of sodium information provided on packaged products and the sodium content were recorded. As secondary analyses, we compared sodium content labelling of products in Kenya by manufacturing location and the sodium content of products available in Kenya and South Africa. A total of 6003 packaged products in 56 food categories were identified. Overall, 39% of products displayed sodium content, though the availability of labelling varied widely between food categories, with coverage in main categories ranging from 0% (yoghurts and yoghurt drinks) to 86% (breakfast cereals). Food categories with the highest median sodium content were herbs and spices (9120 mg/100 g), sauces (1200 mg/100 g) and meat alternatives (766 mg/100 g) although wide variabilities were often observed within categories. Imported products were more likely to provide information on sodium than locally produced products (81% compared to 26%) and reported higher median sodium levels (172 mg/100 g compared to 96 mg/100 g). Kenyan products reported a higher median sodium content than South African products in six categories while South African products had higher median sodium in 20 categories, with considerable variation in median sodium content between countries in some categories. These findings highlight considerable potential to improve the availability of sodium information on packaged products in Kenya and to introduce reformulation policies to reduce the amount of sodium in the Kenyan food supply.

## 1. Introduction

Excess dietary salt intake causes high blood pressure and is associated with increased risks of cardiovascular diseases [1], which are major causes for morbidity and mortality globally and in Africa [2]. Salt is comprised of sodium and chlorine, with the sodium causing elevations in blood pressure. The World Health Organization recommends a maximum intake of 5 g salt (or 2 g of sodium) per day [3], though populations around the world, including in many African countries, exceed this recommended level [4,5]. National dietary guidelines, although often presented in different ways, are generally consistent in recommending that healthy diets include limiting daily sodium intake [6].

The rise in diet-related chronic diseases in Africa is strongly linked to changing structural factors, in particular changes in dietary patterns and increasing consumption of processed packaged foods that are often high in salt, saturated and trans fats and added sugars [7]. In many low-and middle-income countries there has been a substantial change to the food system in recent years, characterized by traditional fresh food markets being replaced by large regional and local supermarkets as the main source of food [8]. In Kenya, the rise in supermarket shopping has led to increased consumption of processed and packaged foods, particularly meat products, bread, pasta, snacks and soft drinks [9]. Increased supermarket purchases have been shown to increase availability of calories per person [9] and are associated with higher body mass index in Kenya [10].

Consumers are increasingly considering the nutritional and health benefits of food products while purchasing [11]. Nutrition labelling is a public health policy tool used to promote healthy dietary patterns through informing consumers about the nutritional quality of packaged food products [12], and may also drive changes in industry practices [13]. Nutrition labelling can take diverse forms and many countries are moving toward mandatory display of nutrition information on pre-packaged food products even in the absence of a nutrition or health claim [14,15]. Kenya has adopted the Codex Alimentarius standard in nutrition labelling [16]. As such, nutrition labelling is mandatory only if a health or nutrition claim is made or the food is for a special dietary use [17]. This regulation also exempts certain foods on the basis of nutritional or dietary insignificance or inadequate space on product packaging. Kenyan standards require a declaration of sodium content in a product’s Nutrition Information Panel (NIP), when one is displayed, and set an upper limit for sodium intake at 2000 mg per day [17].

The World Health Organization advises that food manufacturers should reduce the levels of nutrients of concern, including sodium, in their products to promote healthy diets and reduce non-communicable disease (NCD) burden [18,19]. To date no studies have assessed the completeness of nutrition labelling in Kenya and there have been no previous attempts to quantify the amount of sodium in packaged products in the Kenyan food supply. This is a critical research gap for the development, implementation and evaluation of healthy food environment policies, particularly setting and monitoring nutrition standards, including for the purposes of reformulation. Within this context, the aims of this study were to (1) assess the proportion of packaged foods in Kenya displaying nutrition information for sodium; (2) determine the reported amount of sodium in different packaged food categories in Kenya; (3) compare sodium labelling and reported sodium content in imported and domestically produced products available in Kenya; and (4) compare reported sodium content in Kenyan products to South African products, a country which may share similarities in the food supply due to geographical proximity.

## 2. Methods

This study was a cross-sectional survey of available packaged food and beverage products in Kenya and South Africa.

### 2.1. Data Sources

The sodium content of food and beverage products in Kenya was obtained during data collection carried out by a trained data collection team in June to July 2019. Data was collected from the NIP of all foods and beverages available for sale from one store for each of five large retail supermarkets in Nairobi, Kenya (Tuskys, Chandarana, Kassmatt, Stanmatt and Kamindi Selfridges). Stores were purposively selected, with large supermarkets with several branches preferred as this corresponds with having a larger market share in the Kenyan economy. Under this attribute, Tuskys and Chandarana were chosen as they have 47 and 17 branches, respectively, distributed across Kenya. These two supermarkets are typically located in middle to high income residential areas. To balance with supermarkets from middle to low income residential areas, Kassmatt, Stanmatt and Kamindi Selfridges were also selected.

The sodium content of foods and beverage products in South Africa was obtained from the South African FoodSwitch database. This database contains data collected between 2015 and 2018 through in-store surveys, with a trained data collection team obtaining nutrition information from the NIP of all packaged foods and beverages available for sale from retailers in Johannesburg (Shoprite Checkers, Pick n Pay, Spar and Woolworths). Data was also collected at a national level from crowdsourcing of information reported on food labels, where users of the FoodSwitch app provide photographs of products not identified through the in-store surveys, expanding and updating the database [20].

### 2.2. Data Collection and Processing

The protocol for data entry and quality checks for the databases have been described previously [20] and were consistent across countries. In brief, a smartphone application was used to take photographs and capture product information for all packaged foods including barcodes, product name, ingredients list and the NIP. Product images were sent to a central electronic holding area where a trained research team entered the nutrient data into a database.

Where an identical product (i.e., same product name, nutrition content and package size) was found across multiple stores, only one was included in the analyses. When the same product was available in different pack sizes all were included to represent the diversity of products available on shelves. In addition, where the same product was available in both Kenya and South Africa, it was counted each time for each country.

**Figure 1:**
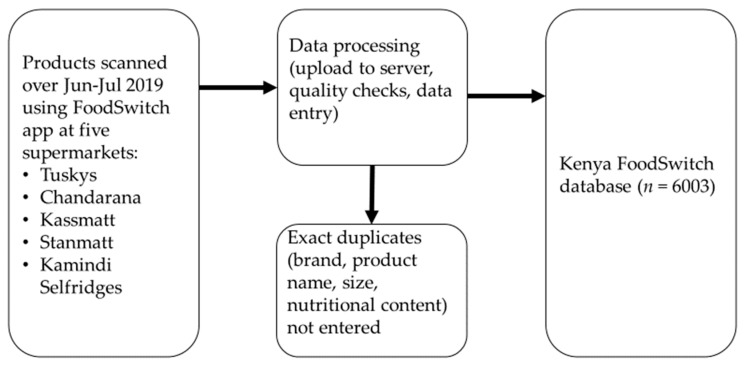
Overview of the development of the Kenya FoodSwitch database, 2019.

An overview of the development of the Kenya FoodSwitch database is provided at Figure 1.

### 2.3. Food Categorisation

Products were categorised in a hierarchical structure into food categories in accordance with the Global Food Monitoring Group categorisation system [21]. This system categorises foods and beverages into food groups then into food categories. An example of a food group is bread and bakery products, while food categories in this food group include breads, biscuits, and cakes, muffins and pastries. Products were also categorised as local or imported depending on the location of the manufacturer as listed on the label. Local products were categorised as those manufactured in Kenya while imported products were manufactured outside Kenya.

### 2.4. Statistical Analysis

Descriptive statistics (medians, interquartile ranges and ranges of sodium levels per 100 g) were calculated and presented for each category of packaged food products available in Kenya. The number and percentage of these products that displayed sodium content information were also reported. The median sodium content of local and imported products, both overall and by food category, and the median sodium content of food categories in Kenya and South Africa were calculated and compared using Mann-Whitney tests. All statistical analyses were conducted using StataIC version 16 (Stata Statistical Software Release 16, 2019; StataCorp LLC, College Station, TX, USA).

## 3. Results

### 3.1. Sodium Labelling and Content of Packaged Food Products in Kenya

A total of 6003 unique packaged foods and beverages across 56 food categories were identified in the Kenyan supermarkets surveyed (Table 1). The food categories with the largest number of products were biscuits (*n* = 412, 6.9% of all products), followed by crisps and snacks (*n* = 401, 6.7% of total), and yoghurt and yoghurt drinks (*n* = 374, 6.2% of total). There were 13 food categories for which ≤10 products were identified.

Overall, 39% of products carried a label describing the sodium content (Table 1). However, the prevalence of sodium labels varied substantially across food categories. Across 21 food categories (including a total of 2351 products), sodium labelling was found on the majority (>50%) of products. Conversely, there were some large food categories (containing at least 100 products) where sodium labelling was rare—including yoghurt and yoghurt drinks (*n* = 374, sodium labelling on 0% of products), herbs and spices (*n* = 329, sodium labelling on 11% of products), other grain and cereal products (*n* = 154, sodium labelling on 7% of products), and cakes, muffins and pastries (*n* = 133, sodium labelling on 11% of products).

**Figure 2:**
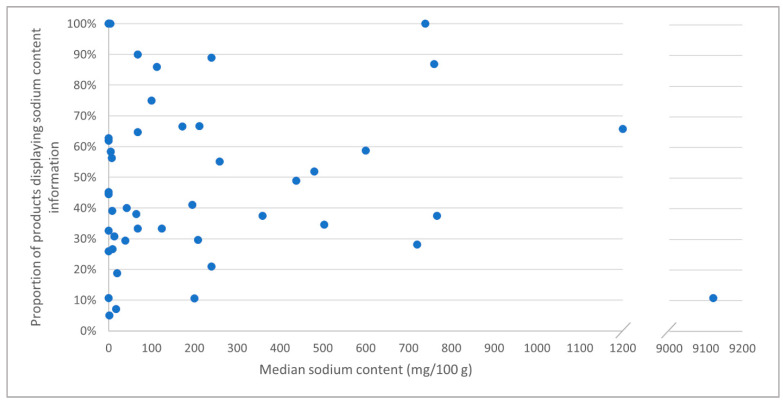
Sodium labelling and content of food categories sampled from five major supermarkets in Nairobi, Kenya, 2019. *n* = 47; nine categories with no products displaying information on sodium content omitted.

The declared sodium content was highly variable between food categories (Table 1; Figure 2). Food categories with the highest sodium content were herbs and spices (median 9120 mg/100 g), sauces (1200 mg/100 g), meat alternatives (766 mg/100 g), noodles (760 mg/100 g), meal kits (739 mg/100 g), cheeses (720 mg/100 g), processed meats (600 mg/100 g) and ready meals (503 mg/100 g). For many of these high sodium categories, information on sodium content was limited; for example, sodium labelling was available only on 11% of herbs and spices (*n* = 36), 38% of meat alternatives (*n* = 6), 35% of ready meals (*n* = 9) and 28% of cheeses (*n* = 40). For several food categories, most notably mayonnaises and salad dressings, the reported median sodium content was 0 mg/100 g.

Our analysis found that the sodium content also varied substantially within food categories (Table 1). Some of the food categories with the largest variation in the sodium content included sauces (8 mg/100 g to 23,437 mg/100 g), herbs and spices (160 mg/100 g to 23,240 mg/100 g), vegetables (0 mg/100 g to 12,000 mg/100 g) and noodles (9 mg/100 g to 11,940 mg/100 g).

### 3.2. Local vs. Imported Products in Kenya

Of the 6003 unique products, a total of 1470 (24%) were imported products and the remainder (*n* = 4533, 76%) were local products (Table 2). Only 26% (*n* = 1171) of the locally manufactured products carried a sodium label, compared to 81% (*n* = 1194) of the imported products. Compared with local products, imported products provided sodium content information across more products in each food category except for noodles and soups.

Overall, the median sodium content of imported products was 172 mg/100 g, significantly higher than locally manufactured products at 96 mg/100 g (*p* < 0.001) (Table 2). The median sodium content was significantly higher in imported products in seven categories, with the greatest differences in median sodium content found in sauces (+870 mg/100 g) and other cereal and grain products (+314 mg/100 g). The median sodium content was significantly lower in imported products in five categories, with the greatest differences in soups (−6060 mg/100 g) and spreads and dips (−243 mg/100 g) (*p* < 0.05 for each).

### 3.3. Comparison to Products in South Africa

There were 46 food categories for which sodium content was available for products in both Kenya (*n* = 2365) and South Africa (*n* = 12,971) (Table S1). Overall, the median sodium content varied between countries in 26 of the food categories assessed (*p* < 0.05).

In six food categories (including cheeses, ready meals, and snacks and dips), median reported sodium content was higher (*p* < 0.05) in Kenyan products compared to South African products, while in 20 categories (including noodles, processed meats, crisps and snacks) the median reported sodium content was higher in the South African products (Table S1). The magnitude of differences appeared substantial in some food categories—for instance, for nuts and seeds, the median sodium content of products in Kenya was ~10.9 fold higher than in South Africa; conversely, in soups, median sodium content in South African products was ~12.6 fold higher than Kenya.

## 4. Discussion

The current study showed that labelling of sodium content on packaged food products in Kenya is limited, with just over one third of products displaying sodium information on the NIP overall and products in several major food categories even less likely to display sodium labelling. Imported products were far more likely to display information on sodium content and were found to have a higher reported average sodium content. The sodium content varied considerably within and between food categories in Kenya and between Kenya and South Africa.

Our findings have important implications for policies and regulations related to food labelling and composition of packaged food products in Kenya. The lack of sodium labelling information makes it difficult for consumers who are concerned about lowering their sodium intake to make informed decisions while purchasing food and beverage products from Kenyan supermarkets. This is particularly problematic for food categories that are high in sodium and yet for which many products do not disclose sodium information, such as herbs and spices, meat alternatives, cheeses and ready meals, as identified in our study; the high sodium categories identified here are largely consistent with previous studies in other countries [22,23,24,25,26,27]). In addition, the absence of sodium labelling will likely hinder efforts to reduce sodium in the food supply since it is difficult for government and other stakeholders to systematically identify high-sodium food categories and monitor sodium reduction efforts [28].

These findings clearly indicate that reformulation to reduce sodium in packaged products is possible and technically feasible. In the absence of data on salt consumption in Kenya [4], reformulation efforts could potentially be targeted toward high sodium categories for which there are demonstrably lower sodium options [29,30]. There may be much to learn from the UK when it comes to implementing a successful reformulation program in Kenya. The early success of the UK targets demonstrates that media pressure, strong government leadership and regular monitoring to assess progress can help achieve success, even under a voluntary scheme [31]. Without such measures, there is little accountability and incentive for food manufacturers to comply with targets [31,32]. Moreover, there must be a commitment to setting meaningful and progressive reformulation targets across a broad range of food categories and with a clear time frame for food industry to act. The experiences of the South African sodium reformulation program [33] and others [34,35,36] suggest that low and/or fixed reformulation targets are unlikely to be enough to effectively reduce sodium consumption in the population.

Kenya’s National Nutrition Action Plan, launched in 2012 [37] and updated in 2020 [38], does not propose nutrition labelling and product reformulation to halt and reverse the prevalence of diet-related NCDs. Furthermore, a recent review of food environment policies in Kenya identified no areas for which government support was assessed as ‘high’ [39]. This review highlighted both the importance and potential of further action, and our findings similarly suggest that mandating nutrition information on packaging, setting nutrition standards (which may include reformulation targets), monitoring food environments and the nutritional quality of the food supply, and using trade policy to control the quality of imported food products may be particularly relevant.

This study is the first to develop and analyse a comprehensive database of packaged food and beverage products available in the Kenyan food supply. It is also the first study to assess the completeness of nutrition labelling in Kenya. Major strengths of our study include the use of contemporary data collected directly from major supermarkets in the largest city in Kenya, enhancing the generalisability of these results to major population centres in Kenya. Data were also collected, processed, and categorised using standardised methods in Kenya and South Africa to ensure that they are comparable; this comparison between countries helps to provide context to the sodium content of foods in Kenya and facilitates attempts to understand what parts of the Kenyan food supply require the most work in terms of sodium reduction.

However, our findings may be less representative of packaged foods and beverages that may be found in rural areas in Kenya, where dietary patterns and food supply may be different from urban areas. A further limitation of the study is the low proportion of products displaying information on sodium content in some food categories. While this is an important finding in its own right, it likely also biases our estimation of the median sodium content for some of the food categories. Comparison of the sodium content between categories within Kenya and also between Kenya and South Africa should therefore be interpreted cautiously. It is possible that our assessment of the sodium content of packaged foods in Kenya could have underestimated the actual sodium content as package labelling might be biased towards products with lower sodium content. Such selective labelling has previously been observed for front of pack nutrition labels [40,41]. The accuracy of the information displayed on the NIP was also not validated, however undertaking such an exercise would require considerable extra resources; this should be the subject of future research. The short data collection period in Kenya (which may not capture potential seasonal variations in the food supply) and the necessary inclusion of different time points for comparisons between countries (2019 for Kenya, 2015-2018 for South Africa) are additional limitations to this study.

Finally, as food environments and dietary patterns continue to change in Kenya, including in rural areas, it is critical that the sodium content of foods and sodium intakes in the population are regularly monitored to examine intake levels and identify major dietary sources of sodium.

## 5. Conclusions

In 2019, just over one third of packaged products in Kenya displayed information about the sodium content. Imported products were more likely to provide information on sodium content than locally produced products and these products were also more likely to report higher median sodium levels. Levels of sodium were high in certain food categories, though the variability of sodium content within food categories indicates the potential for reformulation. These findings have important implications for policy interventions, including mandating nutrition information on packaging (with particular attention to enforcing nutrient labelling on locally manufactured products), setting nutrition standards (which may include reformulation targets), regular monitoring of food environments and the nutritional quality of the food supply, and using trade policy to control the quality of imported food products.

## Figures and Tables

**Table 1:** Sodium labelling and content by category for packaged foods and beverages sampled from five major supermarkets in Nairobi, Kenya, 2019.

Food Group	Food Category	Total Number of Products	Proportion of Products Displaying Sodium Content Information (%)	Sodium Content		
				Median (mg/100 g)	Interquartile Range (mg/100 g)	Range (mg/100 g)
Fruit and vegetables	Herbs and spices	329	10.9	9120	1750–12,010	160–23,240
	Vegetables	251	66.5	172	40–339	0–12,000
	Nuts and seeds	172	20.9	240	8–417	0–3440
	Jams and marmalades	141	32.6	0	0–12	0–8000
	Fruits	87	56.3	7	1–8	0–7397
Dairy	Yoghurts and yoghurt drinks	374	0.0	.	.	.
	Ice creams and edible ices	242	38.0	65	44–116	4–1600
	Milks	177	29.4	39	19–54	12–396
	Cheeses	142	28.2	720	570–820	240–1520
	Creams	18	33.3	68	32–68	20–68
	Desserts	15	26.7	9	0–3009	0–6000
Non-alcoholic beverages	Coffees and teas	358	18.7	20	3–108	0–450
	Fruit and vegetable juices	328	39.0	8	2–24	0–92
	Soft drinks	125	58.4	5	4–12	0–40
	Waters	60	5.0	1	0–15	0–15
	Cordials	28	10.7	0	0–0	0–0
	Energy drinks	10	90.0	68	48–68	0–84
	Electrolyte drinks	5	100	0	0–25	0–40
Bread and bakery products	Biscuits	412	55.1	259	155–390	0–1450
	Breads	169	29.6	209	200–358	0–600
	Cakes, muffins and pastries	133	10.5	200	62–600	0–625
Cereal and grain products	Breakfast cereals	214	86.0	112	12–280	0–1072
	Other cereal and grain products	154	7.1	18	5–320	2–900
	Rice	146	44.5	0	0–0	0–1233
	Pasta	52	30.8	13	1–42	0–1480
	Noodles	38	86.8	760	280–880	9–11940
	Cereal and nut-based bars	6	66.7	212	136–282	72–340
	Cous cous	1	100	4	4–4	43925
Sauces, dressings, spreads and dips	Sauces	345	65.8	1200	392–4160	8–23437
	Spreads and dips	86	48.8	438	249–667	5–3520
	Mayonnaises and salad dressings	51	62.7	0	0–220	0–900
Snackfoods	Crisps and snacks	401	51.9	480	360–665	0–2024
Edible oils and oil emulsions	Cooking oils	157	45.2	0	0–0	0–4
	Edible oils	56	41.1	195	0–630	0–1200
	Coconut oils	8	100	0	0–0	0–200
Meat and meat products	Processed meats	167	58.7	600	70–700	29–2320
	Meat alternatives	16	37.5	766	560–773	520–900
Confectionery	Chocolates and sweets	153	64.7	68	32–102	0–452
	Chewing gums	21	61.9	0	0–8	0–67
	Jellies	4	100	0	0–0	0–0
Sugars, honey and related products	Honeys	108	25.9	0	0–14	0–17
	Dessert additions	25	0.0	.	.	.
	Sugars	21	0.0	.	.	.
	Syrups	5	40.0	42	42–42	42–42
	Dessert toppings	1	0.0	.	.	.
	Sweeteners	1	0.0	.	.	.
Fish and fish products	Processed fish	72	37.5	359	274–391	66–605
Convenience foods	Soups	27	88.9	240	230–1265	40–7240
	Ready meals	26	34.6	503	400–603	309–880
	Meal kits	4	100	739	469–939	338–1000
Special foods	Baby foods	20	75.0	100	80–170	74–215
	Other fitness or diet products	14	0.0	.	.	.
	Sports/protein powders	7	0.0	.	.	.
	Breakfast beverages	1	0.0	.	.	.
Vitamins and supplements	Vitamins and supplements	12	33.3	124	108–487	108–833
Eggs	Eggs	7	0.0	.	.	.
Total		6003	39.4			

**Table 2:** Comparison of sodium labelling and content by category and manufacturing location for packaged foods and beverages sampled from five major supermarkets in Nairobi, Kenya, 2019.

Food Group	Food Category	Local Products			Imported Products			Comparison between Median Sodium Content (*p*-Value)
		Total Number of Products	Proportion of Products Displaying Sodium Content Information (%)	Median Sodium (mg/100 g)	Total Number of Products	Proportion of Products Displaying Sodium Content Information (%)	Median Sodium (mg/100 g)	
Fruit and vegetables	Herbs and spices	305	4.6	7420	24	91.7	9120	0.820
	Vegetables	106	27.4	198	145	95.2	172	0.237
	Nuts and seeds	170	20.6	240	2	50.0	114	0.663
	Jams and marmalades	97	4.1	13	44	95.5	0	0.011
	Fruits	55	38.2	8	32	87.5	4	0.758
Dairy	Yoghurts and yoghurt drinks	367	0.0	.	7	0.0	.	.
	Ice creams and edible ices	161	28.6	110	81	56.8	60	0.472
	Milks	137	12.4	46	40	87.5	31	0.024
	Cheeses	98	4.1	800	44	81.8	710	0.668
	Creams	11	0.0	.	7	85.7	68	.
	Desserts	7	0.0	.	8	50.0	9	.
Non-alcoholic beverages	Coffees and teas	308	15.3	3	50	40.0	108	0.002
	Fruit and vegetable juices	302	37.1	10	26	61.5	3	0.007
	Soft drinks	119	57.1	5	6	83.3	6	0.707
	Waters	59	3.4	1	1	100.0	15	0.221
	Cordials	27	7.4	0	1	100.0	0	.
	Energy drinks	7	85.7	68	3	100.0	68	0.572
	Electrolyte drinks	1	100.0	25	4	100.0	0	0.429
Bread and bakery products	Biscuits	294	42.2	260	118	87.3	250	0.515
	Breads	146	18.5	200	23	100.0	347	0.005
	Cakes, muffins and pastries	122	2.5	200	11	100.0	128	0.696
Cereal and grain products	Breakfast cereals	122	75.4	100	92	100.0	140	0.068
	Other cereal and grain products	137	4.4	7	17	29.4	320	0.018
	Rice	139	43.9	0	7	57.1	0	0.008
	Pasta	10	10.0	1480	42	35.7	5	0.100
	Noodles	15	100.0	760	23	78.3	320	0.468
	Cereal and nut-based bars	5	60.0	224	1	100.0	72	0.180
	Cous cous	1	100.0	4	0	.	.	.
Sauces, dressings, spreads and dips	Sauces	150	40.7	630	195	85.1	1500	<0.001
	Spreads and dips	47	12.8	668	39	92.3	425	0.008
	Mayonnaises and salad dressings	16	12.5	250	35	85.7	0	0.866
Snackfoods	Crisps and snacks	348	46.8	504	53	84.9	360	0.076
Edible oils and oil emulsions	Cooking oils	120	30.0	0	37	94.6	0	0.311
	Edible oils	50	36.0	280	6	83.3	10	0.595
	Coconut oils	3	100.0	0	5	100.0	0	0.439
Meat and meat products	Processed meats	159	56.6	600	8	100.0	820	0.041
	Meat alternatives	10	0.0	.	6	100.0	766	.
Confectionery	Chocolates and sweets	60	26.7	94	93	89.2	64	0.064
	Chewing gums	2	0.0	.	19	68.4	0	.
	Jellies	0	.	.	4	100.0	0	.
Sugars, honey and related products	Honeys	94	22.3	0	14	50.0	15	<0.001
	Dessert additions	25	0.0	.	0	.	.	.
	Sugars	19	0.0	.	2	0.0	.	.
	Syrups	3	0.0	.	2	100.0	42	.
	Dessert toppings	1	0.0	.	0	.	.	.
	Sweeteners	1	0.0	.	0	.	.	.
Fish and fish products	Processed fish	34	14.7	280	38	57.9	360	0.169
Convenience foods	Soups	2	100.0	6300	25	88.0	240	0.020
	Ready meals	17	0.0	.	9	100.0	503	.
	Meal kits	0	.	.	4	100.0	739	.
Special foods	Baby foods	11	54.5	80	9	100.0	165	0.011
	Other fitness or diet products	14	0.0	.	0	.	.	.
	Sports/protein powders	7	0.0	.	0	.	.	.
	Breakfast beverages	1	0.0	.	0	.	.	.
Vitamins and supplements	Vitamins and supplements	4	25.0	833	8	37.5	108	0.157
Eggs	Eggs	7	0.0	.	0	.	.	.
Total		4533	25.8	96	1470	81.2	172	<0.001

## Data Availability

Restrictions apply to the availability of these data. Data were obtained from FoodSwitch (https://www.georgeinstitute.org/projects/foodswitch, accessed on 26 October 2020).

## Supplementary Materials

The following are available online at https://www.mdpi.com/article/10.3390/nu13041385/s1, Table S1: Comparison of sodium content of packaged foods and beverages in Kenya and South Africa by different food categories.
