# Supplementary material for: Sodium Content and Labelling Completeness of Packaged Foods and Beverages in Kenya
Source: Nutrients. 2021 Apr 20;13(4):1385. doi: 10.3390/nu13041385 (PMC8074286; doi:10.3390/nu13041385)
Supplement: Supplementary file 1 [file nutrients-13-01385-s001.zip › nutrients-1185205-supplementary.pdf]

Table S1. Comparison of sodium content of packaged foods and beverages in Kenya and South Africa by different food categories.

Food category	Kenya		South Africa		Comparison between median sodium content (p-value)
	Total number of products	Median sodium (mg/100 g)	Total number of products	Median sodium (mg/100 g)	
Herbs and spices	36	9120	107	6798	0.575
Sauces	227	1200	797	1079	0.971
Meat alternatives	6	766	81	917	0.345
Noodles	33	760	101	1180	0.005
Meal kits	4	739	44	922	0.341
Cheeses	40	720	443	584	0.045
Processed meats	98	600	647	760	<0.001
Ready meals	9	503	314	369	0.025
Crisps and snacks	208	480	454	680	<0.001
Spreads and dips	42	438	261	311	0.027
Processed fish	27	359	359	352	0.523
Biscuits	227	259	687	358	<0.001
Nuts and seeds	24	240	270	22	0.006
Soups	36	240	314	3015	<0.001
Cereal and nut-based bars	4	212	93	148	0.280
Breads	50	209	274	399	<0.001
Cakes, muffins and pastries	14	200	241	335	0.095
Edible oils	23	195	167	400	0.013
Vegetables	167	172	1144	184	0.481
Breakfast cereals	184	112	534	171	<0.001
Baby foods	15	100	258	36	0.041
Chocolates and sweets	99	68	674	63	0.555
Creams	6	68	53	33	0.053
Energy drinks	9	68	85	35	0.089
Ice creams and edible ices	92	65	137	49	<0.001
Syrups	2	42	50	104	0.634
Milks	52	39	434	48	<0.001
Coffees and teas	67	20	212	140	<0.001
Other cereal and grain products	11	18	269	8	0.282
Pasta	16	13	224	5	0.678
Desserts	4	9	102	69	0.108
Fruit and vegetable juices	128	8	851	5	0.064
Fruits	49	7	596	11	<0.001
Soft drinks	73	5	471	8	0.037
Cous cous	1	4	26	10	0.699
Waters	3	1	161	8	0.179

Chewing gums	13	0	46	1	0.308
Coconut oils	8	0	26	2	0.009
Cooking oils	71	0	189	0	<0.001
Cordials	3	0	183	70	0.005
Electrolyte/sports drinks	5	0	38	41	0.004
Honeys	28	0	27	5	0.004
Jams and marmalades	46	0	119	12	<0.001
Jellies	4	0	59	36	0.006
Mayonnaises and salad dressings	32	0	252	464	<0.001
Rices	65	0	97	5	<0.001
